# Reexamination of *Aspergillus cristatus* phylogeny in dark tea: Characteristics of the mitochondrial genome

**DOI:** 10.1515/biol-2022-0838

**Published:** 2024-03-26

**Authors:** Hu Zhiyuan, Chen Lin, Wang Yihan, Dong Meng, Li Yanzi, Xu Zhenggang

**Affiliations:** Hunan Provincial Key Lab of Dark Tea and Jin-hua, School of Materials and Chemical Engineering, Hunan City University, Yiyang 413000, Hunan, China; College of Forestry, Northwest A & F University, Yangling 712100, Shaanxi, China

**Keywords:** *Aspergillus*, *Penicillium*, phylogeny, comparative analysis, dark tea

## Abstract

To enhance our understanding of *Aspergillus cristatus*, an important functional microorganism, the characteristics of its mitochondrial genome were analyzed and compared with related species. The mitochondrial genome of *A. cristatus* was determined to be 77,649 bp in length, with 15 protein-coding regions. Notably, its length surpassed that of the other species, primarily attributable to the intron length. Gene order exhibited significant variations, with greater conservation observed in the genus *Penicillium* compared to *Aspergillus*. Phylogenetic tree analyses indicated that the genera *Aspergillus* and *Penicillium* are closely related but monophyletic. Furthermore, the phylogenetic tree constructed based on protein-coding genes effectively distinguished all strains with high branching confidence. This approach provides a robust reflection of the evolutionary relationship between *A. cristatus* and its related species, offering potential for the development of molecular markers suitable for *Aspergillus* and *Penicillium*.

## 1 Introduction

**Figure 1:**
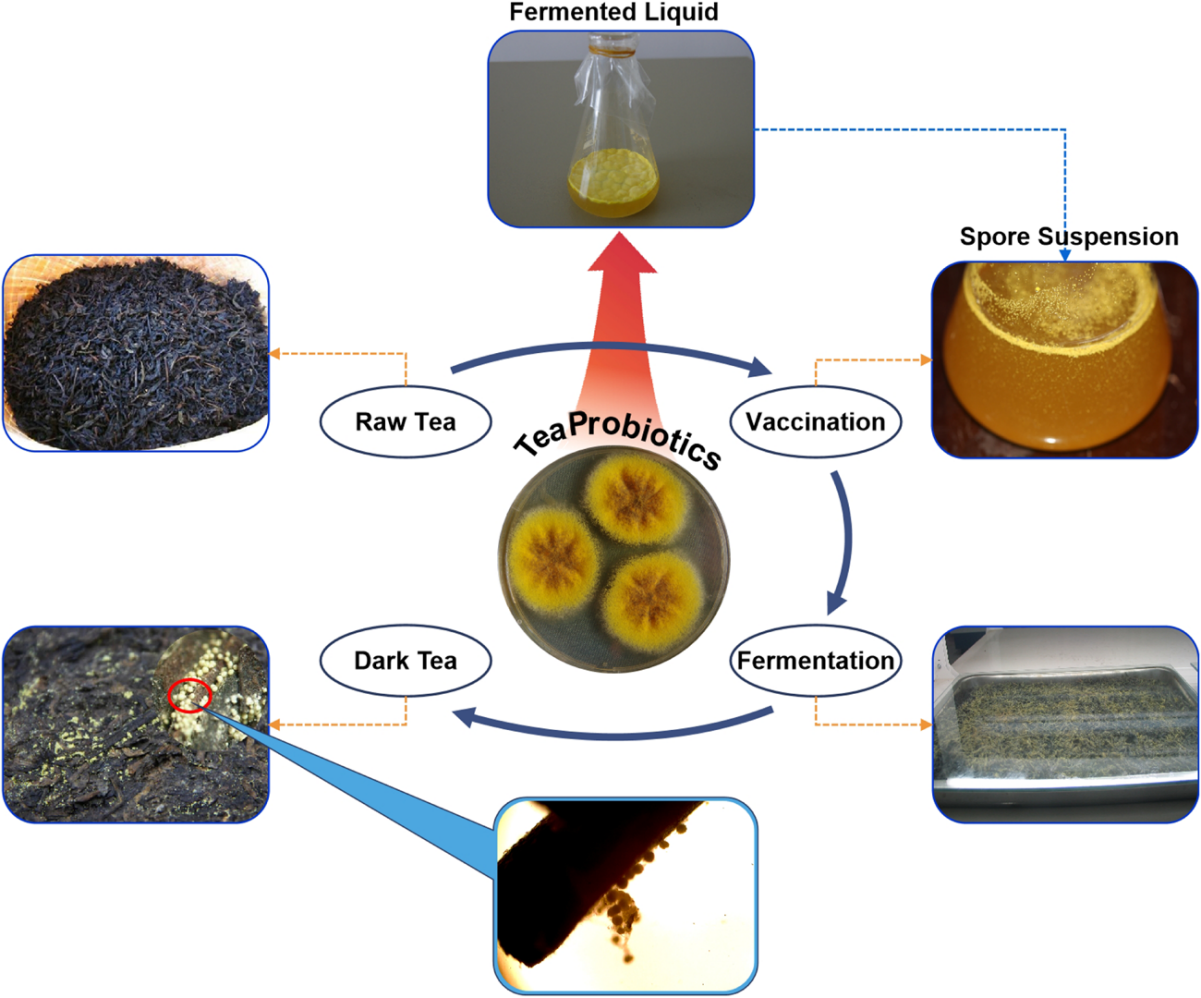
Production process of dark tea vaccinated with *A. cristatus*.

Dark tea, one of the six major types of tea in China, has been shown to exhibit beneficial biological effects, including antioxidant, anti-obesity, anti-diabetic, anti-cancer, cardiovascular-protective, gastrointestinal-protective, hepatoprotective, and other effects [1]. During fermentation, the microbial community composition in dark tea is dynamic and eventually forms a community dominated by *Aspergillus* [2]. *Aspergillus cristatus*, including its sexual morph, *Eurotium cristatum*, is the dominant fungi in dark tea (Figure 1). *A. cristatus* plays a crucial role in enhancing the quality of dark tea, potentially reducing bitterness and astringency while improving the quality and health benefits of the tea [3,4]. In recent years, *A. cristatus* has been utilized as an important functional microorganism; its crude extract at a certain concentration can improve the proliferation and phagocytic ability of macrophages [5] and can be added to various foods or used in fermentation processes with other plants [6,7]. In addition to *A. cristatus*, numerous other *Aspergillus* microorganisms are frequently detected in tea or other fermented foods. Among these, *A. niger* [8,9] and *A. tubingensis* [10] contribute positively to product quality. However, certain *Aspergillus* species such as *A. flavus* [11,12], *A. parasiticus* [13], and *A. parasiticus* [14] have the potential to produce mycotoxins that pose a risk to human health. Therefore, leveraging molecular biology technology for enhanced detection of food microorganisms holds significant importance in improving the safety of fermented food products.

Although considerable research has been conducted on the characteristics of *A. cristatus*, its evolutionary status remains incompletely determined. An analysis of its taxonomic status could uncover previously unknown characteristics. Previously, based on morphological traits, many fungal species in dark tea were assigned to *Aspergillus* [15,16]. With the development of sequencing technology, the evolutionary classification of *Aspergillus* has greatly developed. Through the sequencing of ID regions and partial benA, caM, and *RPB2* genes, species belonging to *Eurotium* were reassigned to the genus *Aspergillus* [17]. Internal transcribed spacer (ITS), 18s rDNA, *RPB2*, calmodulin, beta microtubule protein, or a combination of these sequences are often utilized in the molecular identification of *Aspergillus* but are not completely reliable as taxonomic traits [18]. This suggests that depending solely on one method for species classification is not recommended; auxiliary validation is necessary. The mitochondrial genome is an ideal system for analyzing evolution because of its smaller size [19]. Therefore, it is beneficial to understand the characteristics of the genus to determine the differences in mitochondrial genomes of the same genus. In this study, the mitochondrial genome of *A. cristatus* was analyzed in comparison with *Aspergillus* and *Penicillium* to clarify the evolutionary status of species of *Aspergillus*. Furthermore, a complete understanding of the mitochondrial genome characteristics of *Aspergillus* can also serve as an experimental basis for the development of mitochondrial DNA molecular markers, which holds positive significance for the quality control of dark tea and other fermented foods.

## 2 Materials and methods

### 2.1 Culture and selection of the *A. cristatus* strain

The *A. cristatus* strain JH1209 was obtained from a dark tea sample collected in Yiyang, China (N28°15′, E111°44′). The strain was cultured in different media: PDA, comprising 200 g/L potato, 20 g/L glucose, and 20 g/L agar powder; modified PDA, comprising 300 g/L potato, 80 g/L sucrose, 5 g/L NaCl, and 20 g/L agar powder; CZ20, a variation of the Czapek-Dox medium with an increased sucrose content of 200 g/L; CZ60, a variation of the Czapek-Dox medium with an increased sucrose content of 600 g/L; and CYA, a variation of the Czapek-Dox medium supplemented with 5 g/L yeast extract. Each dish was inoculated with *A. cristatus* and incubated at a constant temperature of 28℃ for 6 days in a temperature-controlled chamber. Thalli exhibiting optimal growth conditions were carefully selected to create fixed specimens, which were examined under a scanning electron microscope (HITACHI S-3000N).

### 2.2 Mitochondrial DNA extraction, sequencing, and annotation

The *A. cristatus* strain was inoculated into a PDL liquid medium and incubated at 28°C for a duration of 5 days. After centrifugation, the culture supernatant was discarded to obtain appropriate bacterial material for subsequent DNA extraction. Mitochondrial genomes were extracted using the DNeasy Mini Kit (Qiagen), and DNA was sequenced using the Illumina HiSeq 2500 sequencing platform in the paired-end mode. The raw sequence data were processed using Base Calling to obtain the sequence data. Quality control of the data was performed using NGS QC [20]. After removing sequence adapters, eliminating connectors, and filtering unpaired, short, and low-quality reads, the high-quality clean data were assembled *de novo* using SPAdes [14].

Clean and high-quality data were obtained and used for *de novo* assembly using SPAdes [21]. The mitochondrial genome of *A. cristatus* was annotated using The MITOS [22], and the annotation results were manually corrected. The annotated mitochondrial genome data of *A. cristatus* were submitted to GenBank under accession number MT457782 [23]. The annotation results were visualized using OGDraw to generate a comprehensive mitochondrial genome map for *A. cristatus* [24].

### 2.3 Mitochondrial genome data collection

The complete mitochondrial genome of 16 fungal species was collected, and *Talaromyces marneffei* was selected as the outgroup. All data were downloaded from the National Center for Biotechnology Information (NCBI; https://www.ncbi.nlm.nih.gov/). The details are given in Table 1.

**Table 1:** Basic information of mitochondrial genome in the research

Species	Length (bp)	GC content (%)	Nucleotide composition (%)				GC skew	Genebank accession	Number of CDS
			A	T	C	G			
*Aspergilluscristatum*	77,649	28.22	37.64	34.14	12.61	15.61	0.1063	MT457782	15
*Aspergillus flavus*	31,602	25.17	36.04	38.8	10.96	14.21	0.1291	NC_026920	14 (lack of *rps5*)
*Aspergillus fumigatus*	30,696	25.48	36.27	38.25	11.20	14.80	0.1385	NC_017016	15
*Aspergillus luchuensis*	31,228	26.42	35.73	37.85	11.86	14.56	0.1022	NC_040166	14 (lack of *rps5*)
*Aspergillus nidulans*	33,227	24.94	37.77	37.29	10.91	14.03	0.1251	NC_017896	15
*Aspergillus niger*	31,103	26.9	35.69	37.41	11.99	14.91	0.1086	NC_007445	14 (lack of *rps5*)
*Aspergillus pseudoglaucus*	53,882	27.81	37.18	35.02	12.47	15.34	0.1032	NC_041427	14 (lack of *rps5*)
*Aspergillus tubingensis*	33,656	26.78	35.95	37.27	12.02	14.76	0.1023	NC_007597	14 (lack of *rps5*)
*Aspergillus ustus*	33,007	25.16	36.36	38.48	10.95	14.21	0.1296	NC_025570	14 (lack of *rps5*)
*Aspergillus parasiticus*	29,141	26.16	35.92	37.93	11.68	14.47	0.1067	NC_041445	15
*Aspergillus oryzae*	29,202	26.15	37.93	35.92	14.44	11.71	−0.1044	KY352472	12 (lack of *rps5,nad4l,atp8*)
*Aspergillus egyptiacus*	66,526	26.54	38.25	35.2	11.51	15.03	0.1326	MH041273	15
*Penicillium citrinum*	27,537	26.81	36.14	37.06	11.83	14.98	0.1175	NC_047444	14 (lack of *rps5*)
*Penicillium digitatum*	28,978	25.34	36.04	38.62	11.37	13.97	0.1026	NC_015080	15
*Penicillium polonicum*	28,192	25.56	35.73	38.71	11.42	14.15	0.1068	NC_030172	14 (lack of *rps5*)
*Penicillium solutum*	28,601	25.47	35.77	38.76	11.42	14.06	0.1036	NC_016187	14 (lack of *rps5*)
*Talaromyces marneffei*	35,438	24.63	36.72	38.65	10.43	14.2	0.1531	NC_005256	—

### 2.4 Analysis of sequence characteristics

The protein coding region, tRNA sequence, and rRNA sequence of the mitochondrial genome of *A. cristatus* were extracted using the Feature Extract 1.2L Server [25]. Sequence composition analysis of each mitochondrial genome was analyzed using BioEdit software [26]. During the analysis, the genome sequences were divided into coding sequence (CDS), tRNA, rRNA, introns, and intergenic regions to compare their differences and characteristics. The search for introns was performed using RNAweasel (https://megasun.bch.umontreal.ca/cgi-bin/RNAweasel/RNAweaselInterface.pl) [27].

### 2.5 Relative synonymous codon usage (RSCU)

After artificially removing introns from the protein-coding sequence, DAMBE was employed to calculate the types and proportions of amino acids encoded by mitochondrial protein-coding genes of *A. cristatus* and its related species [28]. Additionally, the RSCU was analyzed to understand the codon usage pattern. Stacking and heat maps were produced using the R software (https://www.r-project.org/) [29].

### 2.6 Mitochondrial genome comparison

BRIG [30] software was used for sequence conservation analysis, with *A. cristatus* as a reference. To explore further details of the rearrangement events, the mitochondrial genome sequences of *A. cristatus* and 16 related species were compared and analyzed using the online software MAUVE [31]. Initially, the “Alignsequences” option was selected, and the Genbank format file was submitted. Subsequently, we identified gene rearrangements in the mitochondrial genome through collinear analysis of the genome annotation sequences. The Ka/Ks value was then calculated to show the evolution pattern, using the *A. cristatus* protein-coding sequence as the reference sequence and DNAsp software [32].

### 2.7 Phylogenetic analysis

The mitochondrial sequence of *A. cristatus* was submitted to the NCBI and compared with the Nucleotide database. Subsequently, 29 fungi exhibiting a higher relative degree of similarity to *A. cristatus* were selected for phylogenetic analysis. After concatenating the 12 common protein-coding genes from these fungal mitochondrial genomes in the order *cob-nad1-nad4-atp8-atp6-nad6-cox3-cox1-atp9-nad3-cox2-nad4L-nad5-nad2*, sequence comparison was conducted using MEGA 7.0 [33] software, and the resulting phylogenetic tree was generated using Maximum likelihood methods.

## 3 Results and discussion

### 3.1 Colony morphology and microscopic characteristics

**Figure 2:**
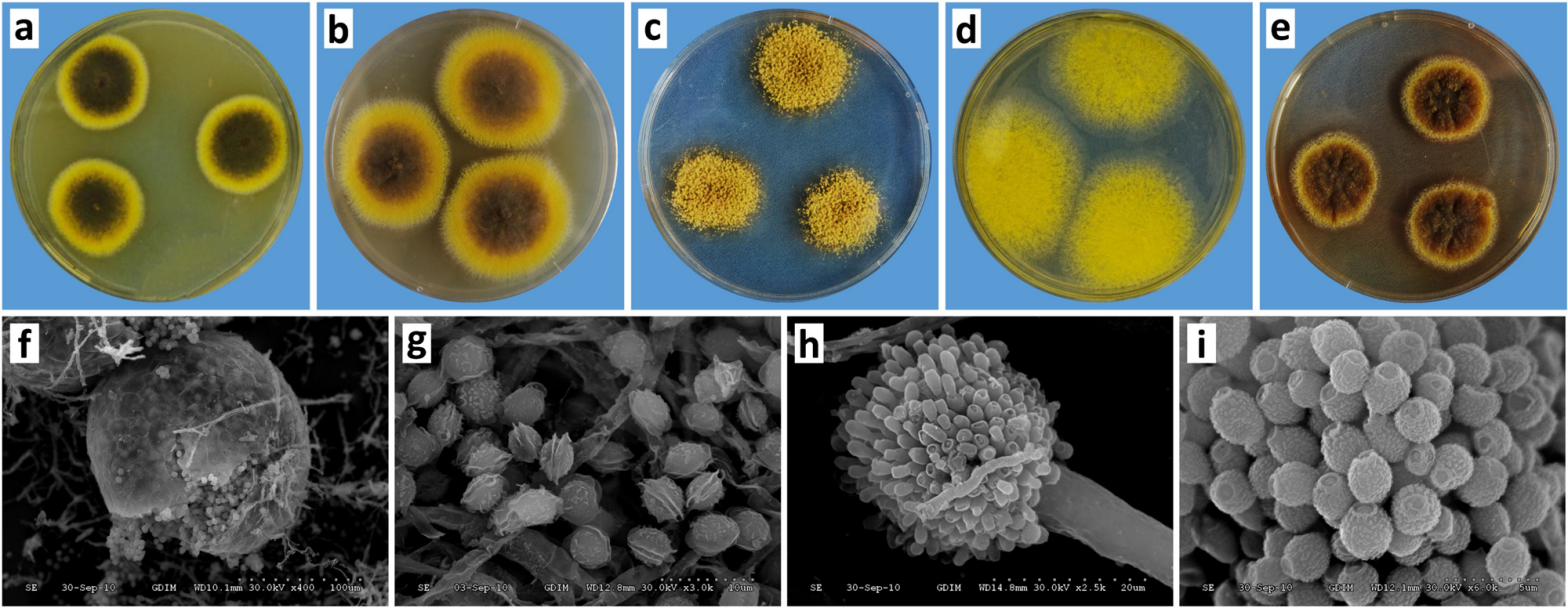
*A. cristatus* colonies and microscopic characteristics: (a) PDA, (b) modified PDA, (c) CZ20, (d) CZ60, (e) CYA, (f) ascocarp, (g) ascospore, (h) conidia head, and (i) conidia.

*A. cristatus* grew successfully on various media, including PDA, modified PDA, CZ20, CZ60, and CYA (Figure 2a–e). Asexual colonies formed on CZ60, whereas the other media induced the production of sexual colonies dominated by ascospores. This suggests that high osmotic pressure triggers sexual reproduction in *A. cristatus*, which is consistent with the findings of Ge et al. [34].

Under the scanning electron microscope, the sexual structure of *A. cristatus* appeared as a spherical cleistothecium, measuring 50–120 µm in diameter (Figure 2f). The ascospores had a size range of 3.7–4.5 µm × 4.4–6.0 µm, exhibiting a rough surface with small pores. Notably, the spores displayed two distinct “coronal” processes when viewed from the equatorial perspective (Figure 2g). On the other hand, the asexual structure of *A. cristatus* consisted of a conidial head measuring 50–80 µm in length (Figure 2h), with each conidial chain containing 3–4 conidia. The conidia themselves were ellipsoidal, with dimensions of 3.4–3.7 µm × 4.2–4.8 µm, and featured multiple irregular verrucous processes on the surface (Figure 2i). Phenotypic differences can serve as a supplementary basis for the identification of *A. cristatus* and other *Aspergillus* species; however, certain limitations exist. For instance, variations in colony morphology may occur due to disparities in culture medium and duration, and microstructural dissimilarities may not be sufficiently distinctive among closely related species [35]. Therefore, further assessment through molecular biology techniques is necessary.

### 3.2 Characteristics of *Aspergillus* mitochondrial genomes

Among the 17 microorganisms analyzed in this study, the total genome lengths ranged from 27,537 to 77,649 bp. Notably, the mitochondrial genome of *A. cristatus* (77,649 bp) was considerably longer than that of other species. This difference is speculated to be because *A. cristatus*, as the dominant strain of dark tea fermentation, underwent many mutations in mitochondrial DNA under the influence of the artificially created special environment, forming intron and accessory genes of considerable length [36]. The GC deviation of the 17 strains ranged from 0.1022 to 0.1531, with a large gap in the base deviation value. According to previous studies [37,38], there is a certain relationship between base deviation and the environmental adaptation of species. This suggests that each fungal strain faces different environmental pressures. *A. cristatus*, *A. fumigatus*, *A. nidulans*, *A. parasiticus*, and *P. digitatum* have 15 CDS regions. The other nine species were found to lack the *rps5* gene, except *A. oryzae*, which lacked three genes: *nad4L*, *rps5*, and *atp8* (Table 1).

**Figure 3:**
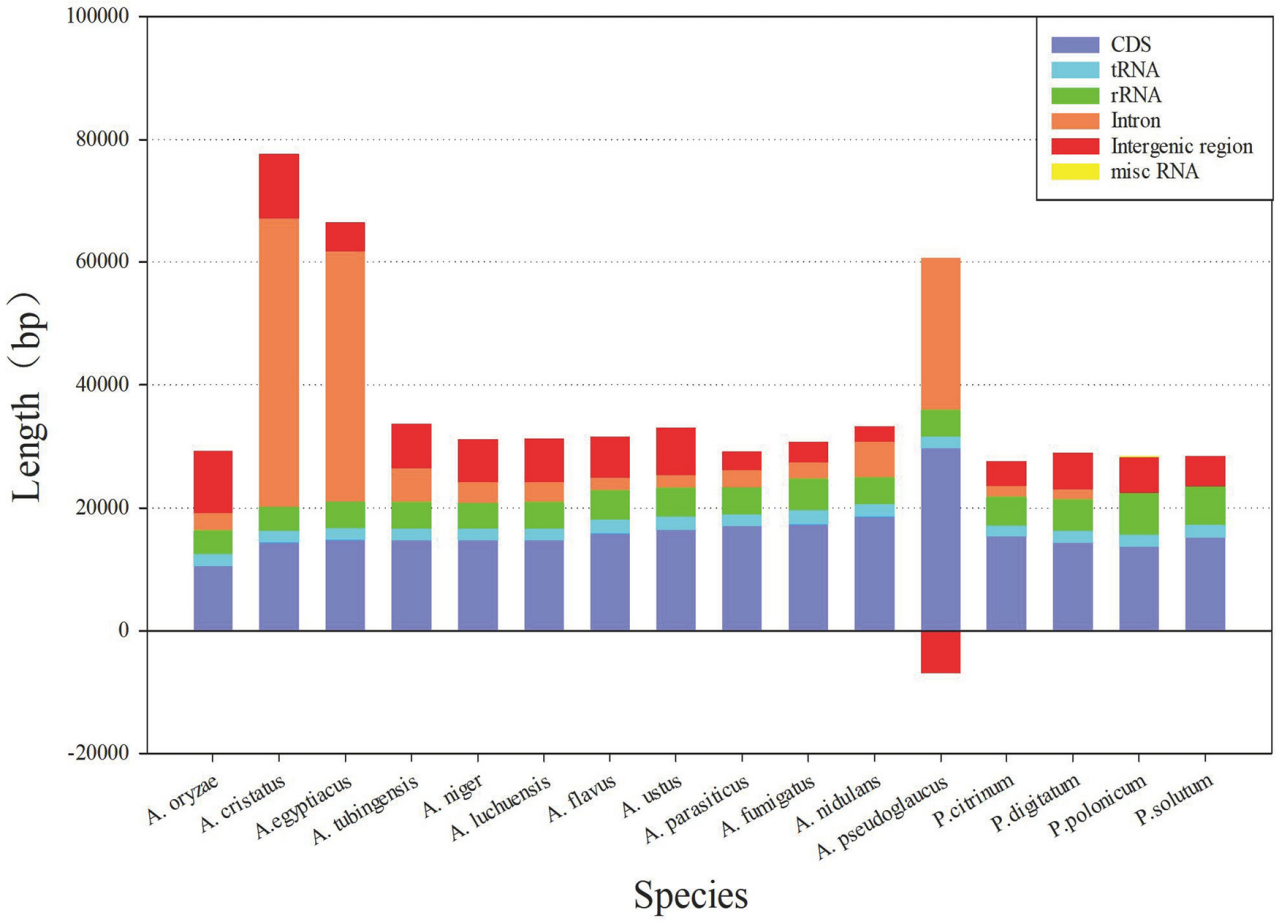
Length of composition sequence for each mitochondrial genome (the negative stack of intergenic region in *A. psedoglaucus* is caused by hypothetical gene’s insertion).

**Figure 4:**
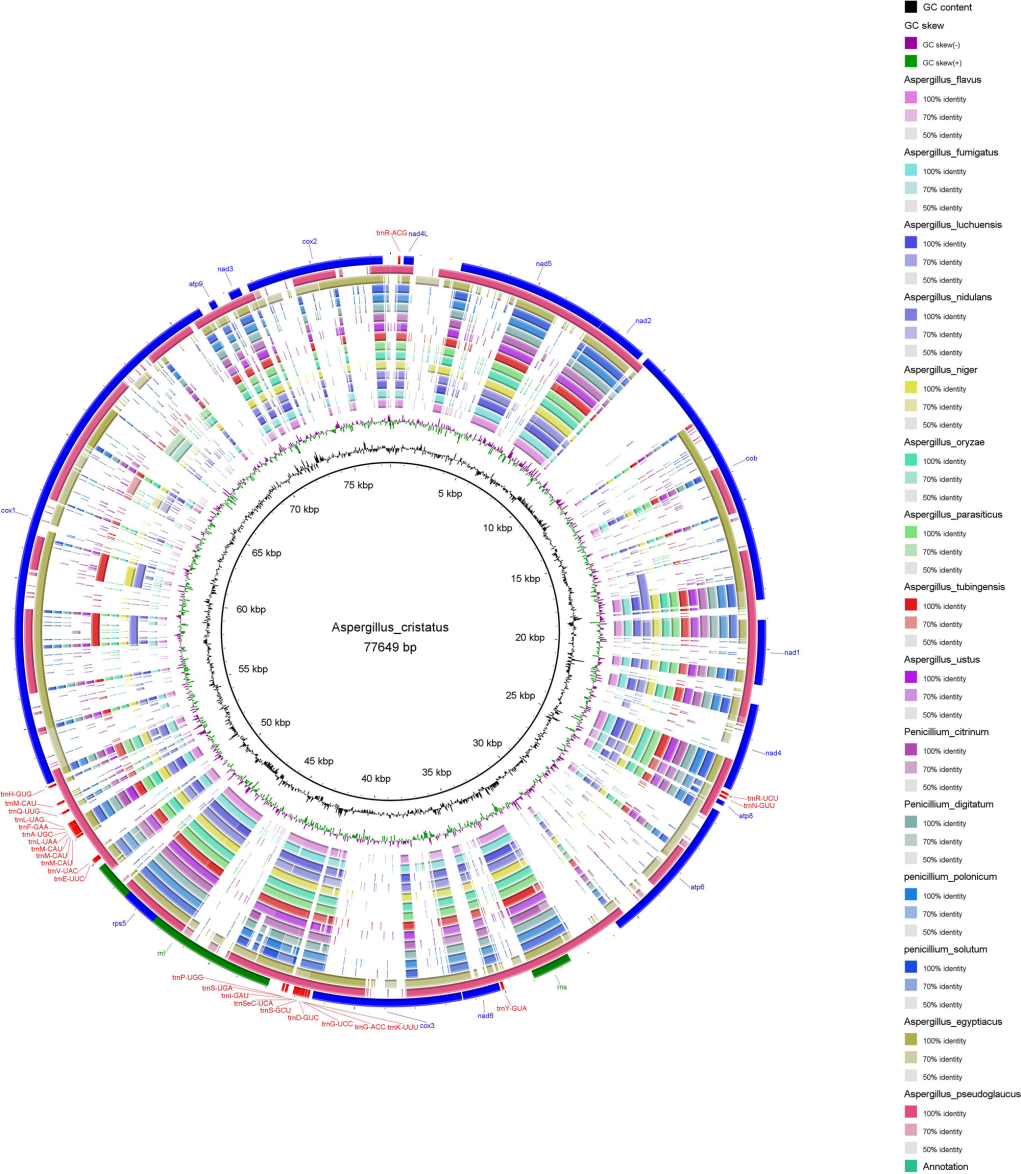
Comparative analysis of conserved region in selected species. The inner black circle line is the reference genome of *A. cristatus*.

Fungal mitochondrial genomes vary greatly in length and composition [19]. The difference in total genome length is mainly reflected in the length of the introns (Figure 3). The intron regions of *A. cristatus*, *A. egyptiacus*, and *A. pseudoglocus* were significantly longer than the corresponding regions in the other species. The similarity of the above three species is not only reflected in the length of intron regions but also in the length of conserved regions, which are longer than those of the other species (Figure 4). It is also worth mentioning that the length of the CDS regions in *A. pseudoglocus* is significantly larger than that in other species because of the large number of open reading frames annotated in its genome, most of which encode endonucleases [39]. The regions with stable lengths were tRNA (approximately 70–80 bp) and rRNA (approximately 4,000–6,000 bp), whereas the ribonuclease pRNA (misc RNA) appeared only in *P. polonicum*. RNA genes in the species studied in this work occurred in a fixed cluster form, tRNA cluster I-rnl-tRNA cluster II, as mentioned in previous literature [40].

### 3.3 Analysis of RSCU

**Figure 5:**
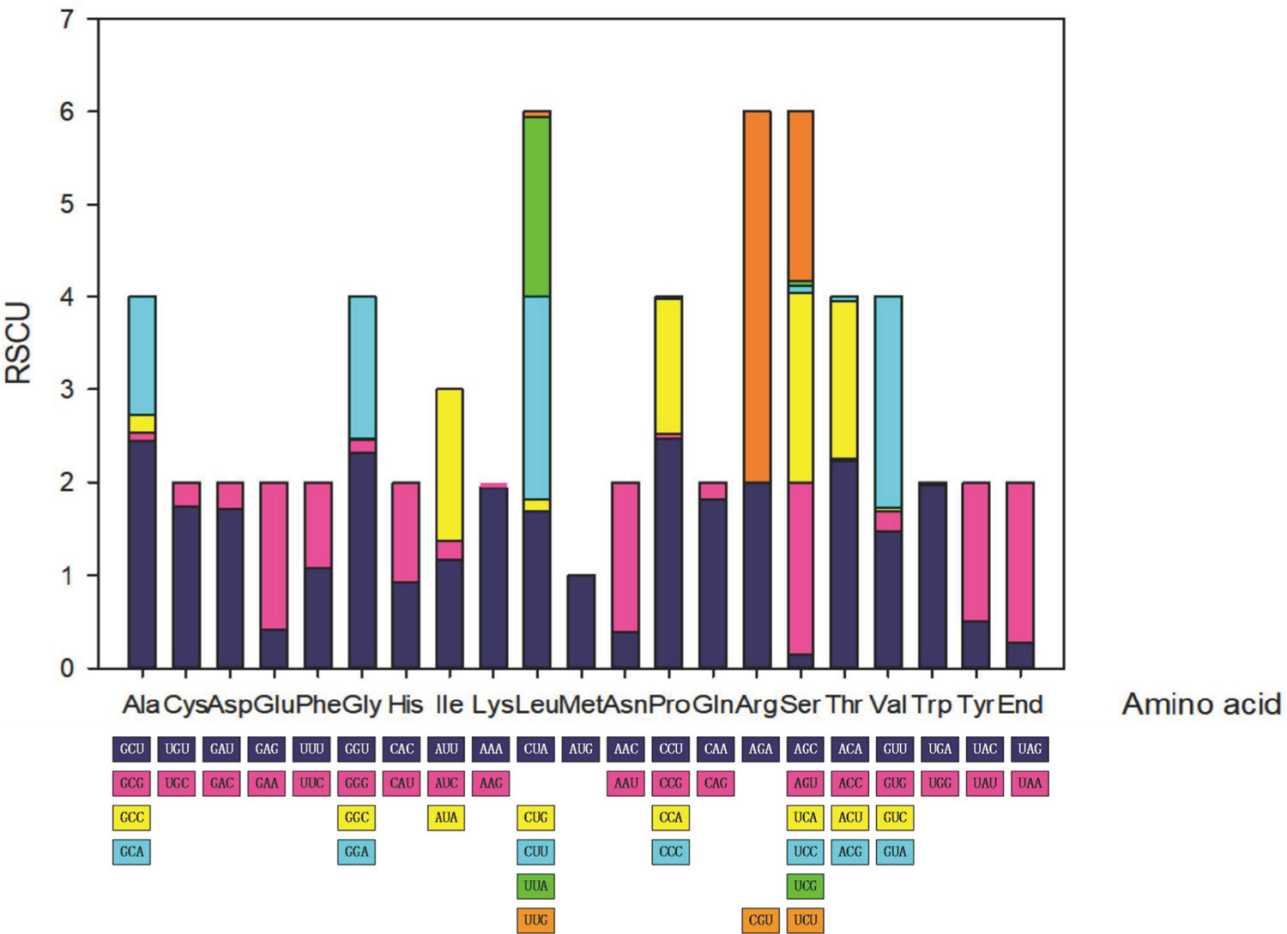
RSCU in *A. cristatus*.

**Figure 6:**
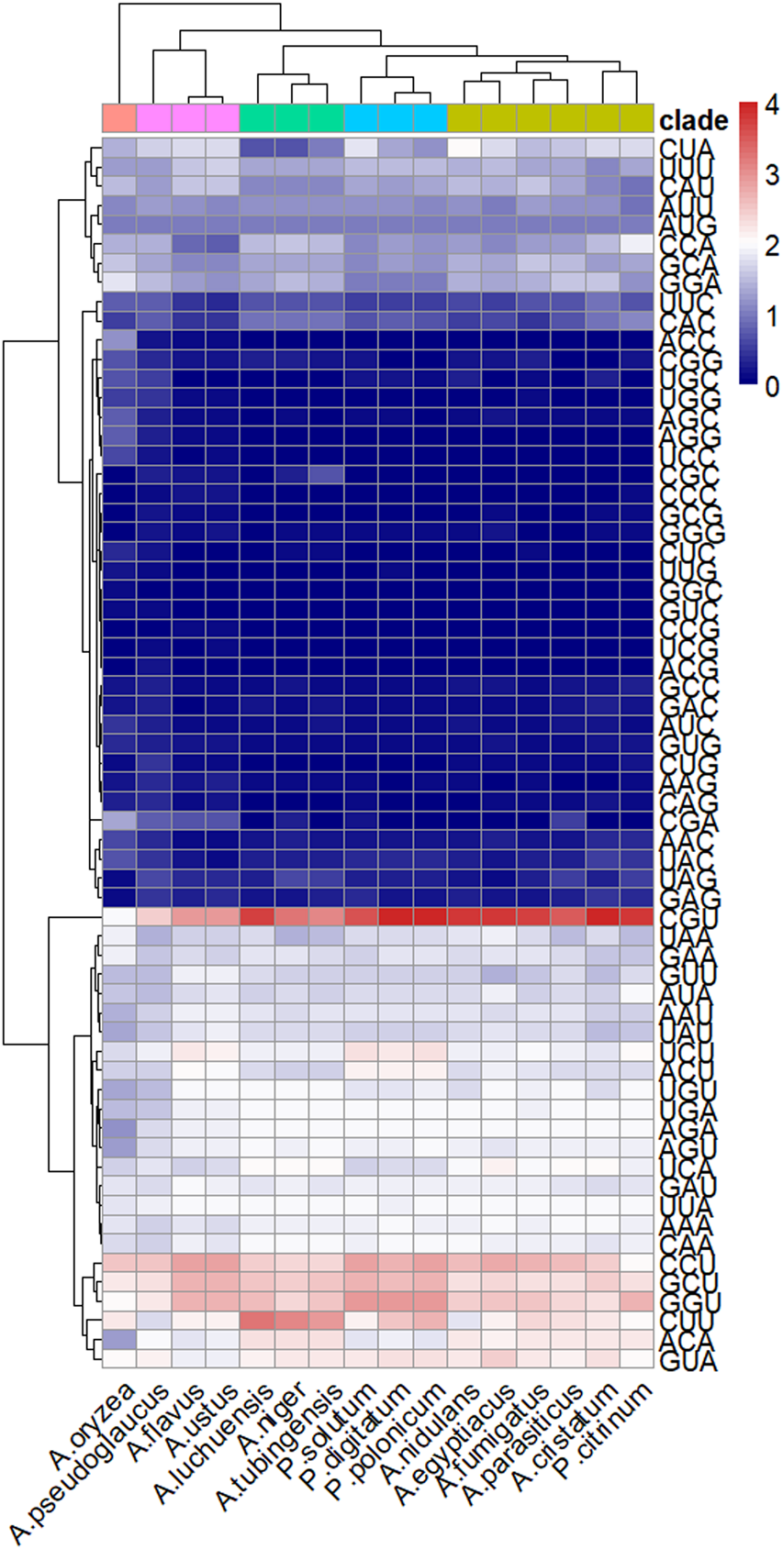
The heat map of RSCU in *Aspergillus* and *Penicillium* species.

Through the analysis of *A. cristatus* codons, we found 31 codons with RSCU values greater than 1 and 32 codons with RSCU values less than 1. Codons corresponding to Phe and His had RSCU values close to 1 and no obvious bias. Except for these two amino acids, the other amino acids showed a more obvious association and contained at least one optimal codon. Although arginine corresponds to six codes, only two types (AGA and CGU) are evident in *A. cristatus*. Codons with third positions A and U were more likely to be preferred, whereas those with third positions G and C were not selected. This may have resulted in a decrease in the GC content of protein-coding sequences (Figure 5). The RSCU values of *A. cristatus* and *P. citrinum* showed the highest similarity (Figure 6), with approximately half of the codon pairs having RSCU values below 1, indicating their relatively lower frequency of usage [41]. Among all the codons, the codon CGU encoding arginine appeared very frequently in all species, whereas the RSCU values of other codons encoding arginine (AGA, AGG, CGA, CGC, and CGG) differed by species but were all close to or equal to 0.

### 3.4 Evolutionary pattern of *Aspergillus* and *Penicillium*

**Figure 7:**
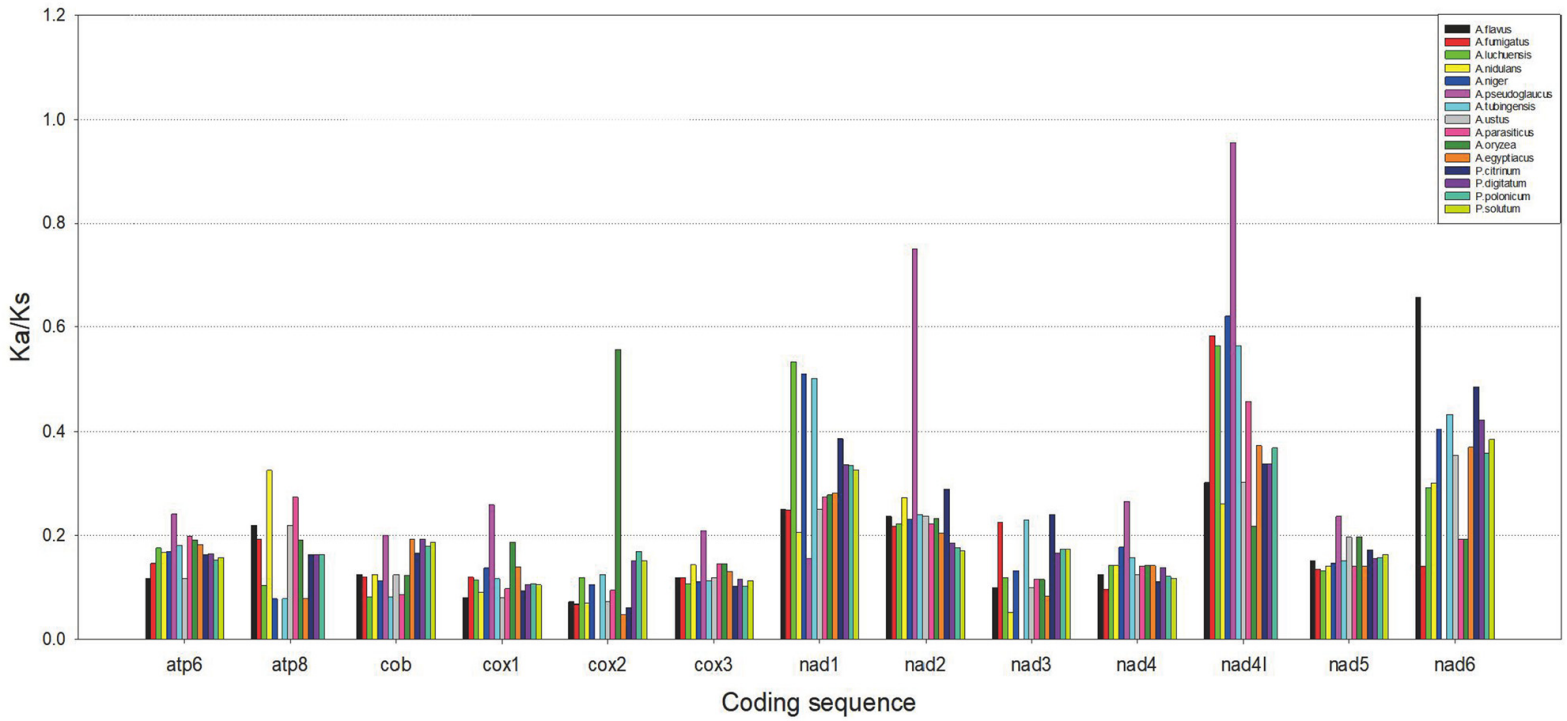
The Ka/Ks ratios of homologous protein-coding genes for *Aspergillus* and *Penicillium* species with *A. cristatus* as the reference.

To better understand the evolutionary pattern, it is necessary to calculate Ka/Ks, which is the ratio of the number of non-synonymous substitutions per non-synonymous site to the number of synonymous substitutions per synonymous site [42]. The Ka/Ks values of all core proteins were less than 1, most of which were <0.4 (Figure 7). The values of core proteins were stable among almost all species studied, but the values of a few genes (*cox2*, *nad2*, *nad1*, *nad4L*, and *nad6*) were unstable across all species. The highest value was for the *nad4L* gene in *A. pseudoglaucus*, although it was still less than 1. The Ka/Ks values of most of the protein-coding sequences of the studied species were close to 0, indicating that all of the protein-coding genes were evolving under strong purifying selection in these species [43,44] In addition, syntenic analysis illustrated that the similarity was very general and the similarity rate was higher in species with similar lengths.

### 3.5 Gene rearrangement in *Aspergillus* and *Penicillium* species

**Figure 8:**
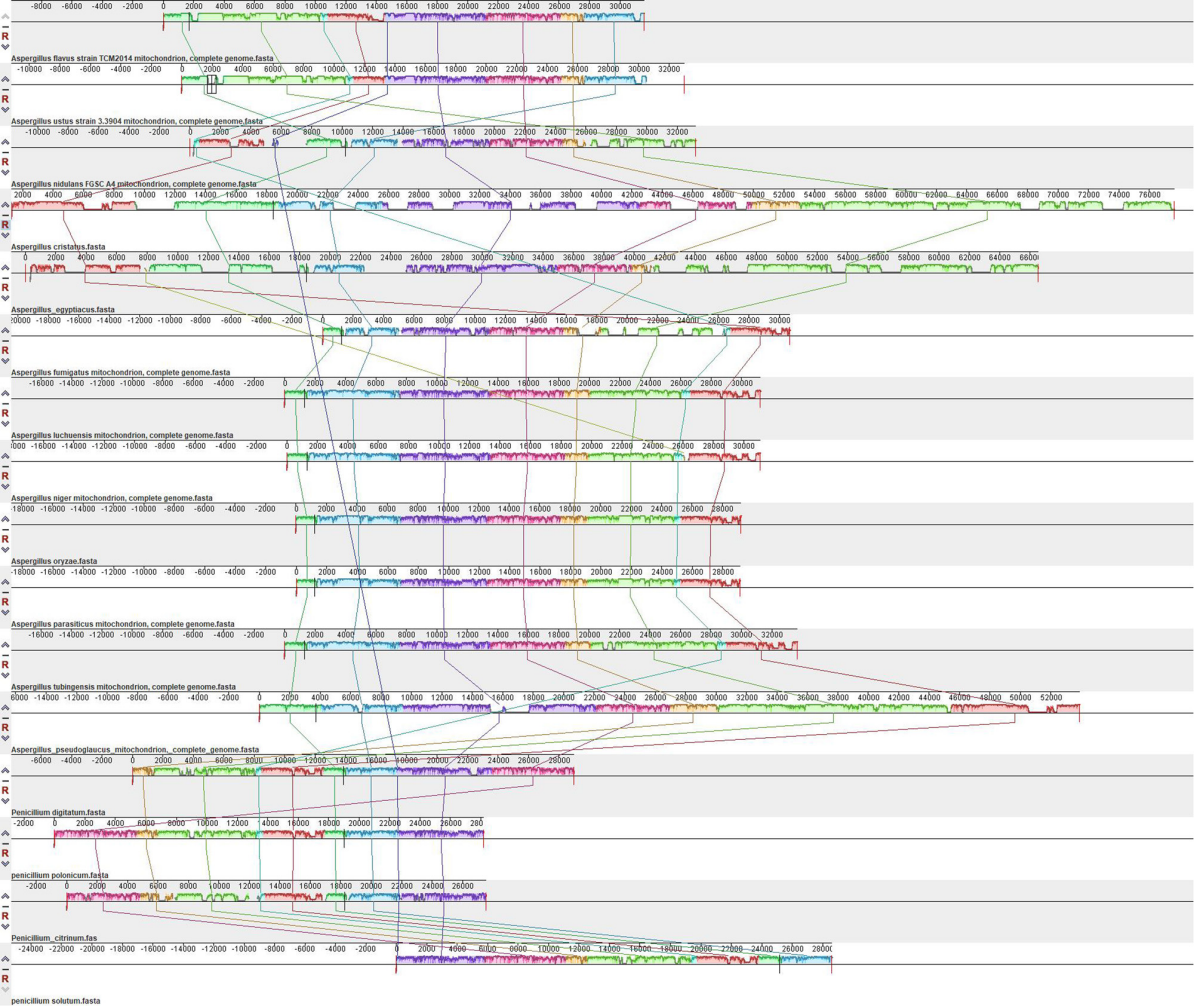
The rearrangement of mt genome of selected species (the similar sequence regions have been classified into the same color stripe).

Gene rearrangements are important in phylogenetic and evolutionary analyses. The positional relationships among the selected species are shown in Figure 8. Overall, the gene sequence of the species studied was stable: *cob*-*nad1*-*nad4*-*atp8*-*atp6*-*rns*-*nad6*-*cox3*-*rnl*-*cox1*-*atp9*-*nad3*-*cox2*-*nad4L*-*nad5*-*nad2*, the genetic sequence conforms to the typical pattern observed in *Aspergillus* fungi [45]. Slight differences were observed in *A. ustus* and *A. flavus*, primarily manifesting in the translocation of two gene junctions, *atp8*-*atp6*-*rns*-*nad6*-*cox3*-*rnl* and *cox1*-*atp9*-*nad3*-*cox2*. In addition to the translocation of gene junctions, there were also translocations of single genes, such as *nad2*, in these two species.

In addition to the rearrangement of protein-coding genes, tRNA also appeared, and *tRNA-Asn*, *tRNA-Ile*, and *tRNA-Ala* in *A. fumigatus* and *tRNA-Pro* in *P. citrinum* all underwent inversion (from the positive strand to the negative strand); however, this was not a common phenomenon in these species. Predominantly, tRNA occurred in the form of clusters together with rRNA and was broken by short AT-rich sequences, which may play an important role in their integral transcription [40].

### 3.6 Phylogenetic tree of *A. cristatus*

**Figure 9:**
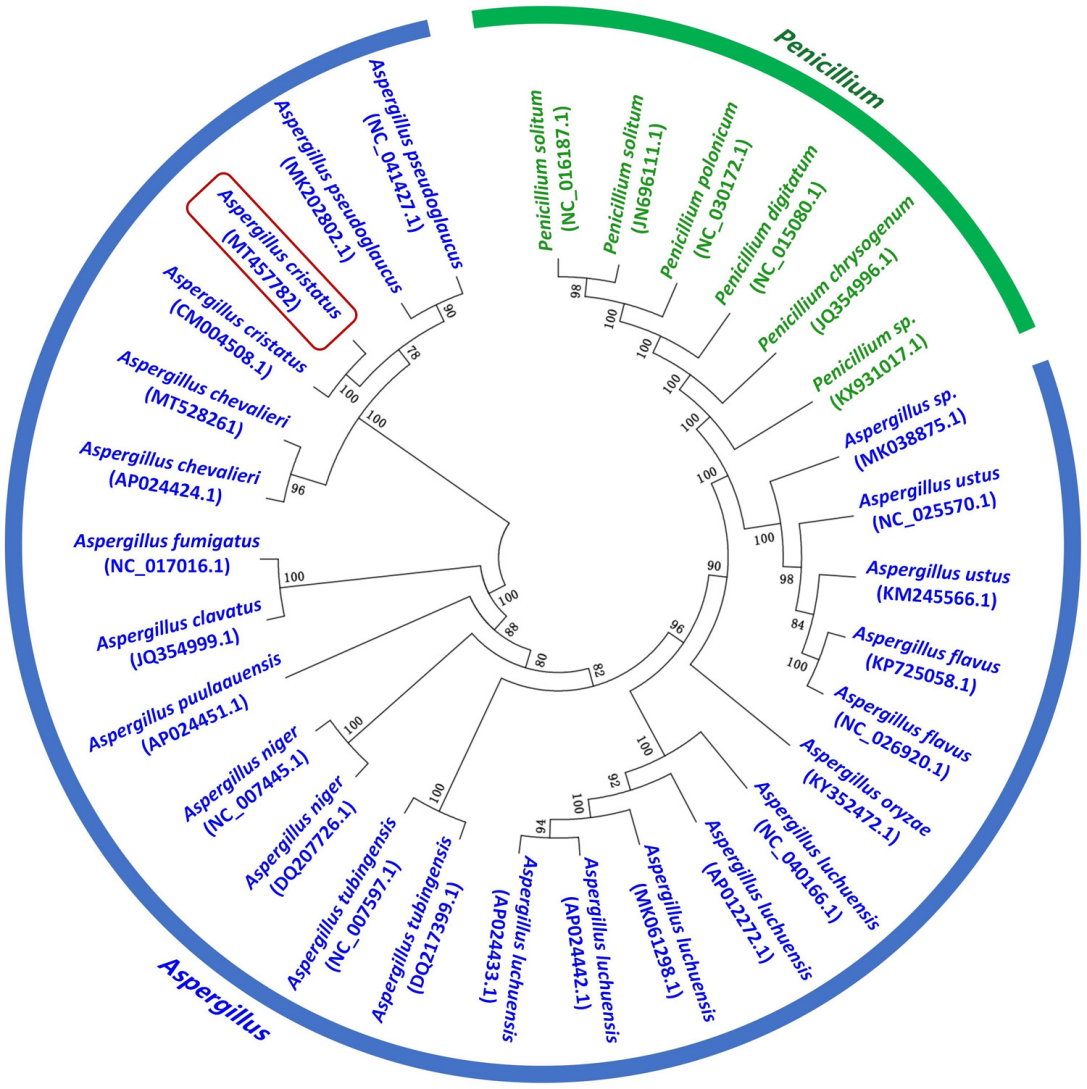
Phylogenetic relationships based on the conserved protein-coding sequences with the Maximum likelihood method.

The phylogenetic tree (Figure 9) reveals that *A. pseudoglaucus* is the closest relative to *A. cristatus*, followed by *A. chevalieri*, which forms a compact cluster. According to the research conducted by Wang et al. [16], *A. pseudoglaucus* was isolated from a new variety of dark tea produced in Guizhou, China. This fungus exhibited similarities to *A. cristatus* in colony morphology and spore characteristics, and *A. pseudoglaucus* produced abundant extracellular enzymes, promoting fermentation [46]. The resemblance between the two fungi often led to confusion, and it was speculated that they shared a similar genetic background but diverged at a later stage.

Furthermore, each branch in the phylogenetic tree exhibits a high confidence level, providing robust support for the phylogenetic relationship between *Penicillium* and *Aspergillus* species. Chen et al. [47] constructed a phylogenetic tree using the Bayesian inference method based on PCGs + rRNA, while Asaf et al. [48] employed Maximum likelihood and Bayesian inference methods to construct a phylogenetic tree based on the complete mitochondrial genome. Both studies reached similar conclusions to those presented in this study. For instance, *A. flavus* and *A. ustus* clustered into a adjacent branch, and *A. tubingensis* and *A. niger* clustered into another adjacent branch. Additionally, closely related species, such as *P. solitum*, *P. polonicum*, and *P. digitatum* were identified. These findings highlight the stability and reliability of mitochondrial genome information in accurately reflecting interspecies relationships among diverse microorganisms.

## 4 Conclusion

Compared to species identification technology based on ITS sequencing, mitochondrial genome information offers greater accuracy and comprehensiveness in species classification and identification. It can provide highly credible insights into variations in gene structure and arrangement order among different species. Currently, mitochondrial genome sequencing technology is widely employed in studying species origin, genetic differentiation, interspecies relationships, population genetic structure, and other related fields. In this study, we isolated and cultured *A. cristatus* from dark tea to determine and analyze the complete mitochondrial genome sequence. The research findings further unveil the phylogenetic position of *A. cristatus* and their associated species.

By analyzing the mitochondrial sequences of species of *Aspergillus* and *Penicillium*, it was found that fungal mitochondrial sequences are substantially distinct in length and composition. The fungal mitochondrial genome length varies considerably [19], and most contributors to the sequence length are intron regions. The phenomenon of varying intron lengths reported in the present study agrees with the findings of Joardar et al. [49]. In terms of composition, most introns in *A. cristatus* were annotated as group I introns, group I are derivatives of self-splicing RNA enzymes (ribozymes), which exist in rRNA, tRNA, and protein-coding regions, and are considered mobile elements that increase the possibility of gene recombination [50]. We observed relatively few gene rearrangements between *Aspergillus* and *Penicillium*, suggesting that the gene order is comparatively conserved in closely related species, irrespective of the number of intron regions. A typical characteristic is that gene rearrangements often appear in the form of clusters and seldom as single genes. Similar phenomena were observed in *A. ustus* [51], in which two clusters were found to be translocated: *nad1*-*nad4* and *cox1*-*atp9*-*nad3*-*cox2*-*nad4L*-*nad5*. These patterns can be elucidated through a tandem duplication/random loss model.

Introns are commonly found in conserved mitochondrial genes across fungal mitochondrial genomes [52]. Notably, the *A. cristatus* cob gene harbors an unusual type II intron, as identified through BLAST analysis in the *Annulohypoxylon* genus. Thus, introns in *A. cristatus* might be frequent in horizontal gene transfer, not only limited to fungal species but possibly communicated between fungi and terrestrial plants. The two genera, *Penicillium* and *Aspergillus*, are closely related, as confirmed by the results we report here; the genera formed good monophyly based on the analysis of mitochondrial sequences of protein-coding sequences, suggesting that it is a feasible method to evaluate the evolutionary pattern of species of *Aspergillus*. However, the phylogenetic relationship between the two genera is still controversial, with one perspective being that they are monophyletic and another that they are paraphyletic. The “monophyletic” opinion, as proposed in our research, is based on the well-defined phylogenetic tree; only *P. citrinum* emerges as notably distinct from other *Penicillium* species. However, it has been suggested that *A. oryzae* is a member of the monophyletic group *A. flavus*, although it does not have a consistent phenotype, which may be the result of strong selection associated with domestication [53]. However, the mitochondrial genomes of *A. flavus* and *A. ustus* showed a high degree of similarity, indicating that the nuclear and mitochondrial genomes most likely evolved separately.

Morphological characteristics are important for the identification and classification of *Aspergillus* and *Penicillium*. Tsang et al. discussed the presence or absence of a conidial head structure and the effects of mutations on conidial morphology and taxonomy [54]. However, morphological identification is usually related to nomenclature; in naming *Aspergillus* species one is faced with a choice between sexual and asexual nomenclature, which has led to occasional misclassification (previously) of some *Aspergillus* species exhibiting no sexual morphology. John et al. suggested grouping some species that are closer in morphology and phylogeny in a subset called “narrow” *Aspergillus*. Phylogenetic analysis revealed that the phylogenetic tree constructed by mitochondrial DNA protein-coding gene could effectively distinguish all strains with a high branching confidence, which could better reflect the evolutionary relationship between *A. cristatus* and its related species, and could be used for further development of DNA molecular markers suitable for *Aspergillus* and *Penicillium*. However, the study has some limitations. The reference sequences for fungal mitochondrial DNA mainly come from relevant databases, and the reliability of specimens will directly affect the results of the study, introducing constraints to fungal identification based on sequence alignment. Moreover, the number of *Aspergillus* species published mitochondrial genome information is currently limited. More accurate and comprehensive conclusions need to be supported by more sequencing data in the future.

In summary, we have elucidated the mitogenomic characteristics of *A. cristatus* and conducted a comparative analysis with closely related species to propose a probable evolutionary position. The results provided a crucial foundation for elucidating the taxonomic status of *A. cristatus* in dark tea and also served as a basis for further investigating the phylogenetic relationship between *Aspergillus* and its related species. Moreover, employing molecular biology technology to accurately monitor the microbial population composition and changes during the fermentation process of dark tea is conducive to improving fermentation technology in tea production, thereby enhancing the quality of dark tea products and ensuring microbial safety.
